# Will land circulation sway “grain orientation”? The impact of rural land circulation on farmers’ agricultural planting structures

**DOI:** 10.1371/journal.pone.0253158

**Published:** 2021-06-24

**Authors:** Jiquan Peng, Juan Chen, Chen Su, Zhifeng Wu, Liu Yang, Wenji Liu

**Affiliations:** 1 School of Economics, Jiangxi University of Finance and Economics, Nanchang, China; 2 School of Business Administration, Zhongnan University of Economics and Law, Wuhan, China; Institute for Advanced Sustainability Studies, GERMANY

## Abstract

This study calculates the effect of different types of land circulation on farmers’ decision-making regarding agricultural planting structure, using field survey data involving 1,120 households in Hubei province, China, and PSM (propensity score matching) and GPSM (general propensity score matching) methods. Results from PSM showed that land circulation could significantly increase farmers’ decisions to plant food crops, which confirms the positive effect of rural land circulation on the “grain orientation” of crop planting structure. Results from GPSM further indicate that the total land circulation, the paddy land circulation, and the dry land circulation all have significantly positive effects on planting structure adjustment towards “grain orientation.” Additionally, planting structure adjustment towards “grain orientation” increases as the scale of land circulation increases, and the former shows a higher rate of increase than the latter, which confirms that rural land circulation facilitates an adjustment in structure towards planting food crops.

## Introduction

The issues arising from the loss of cultivated land, such as cultivated land fragmentation or abandonment, cultivated land outflow toward construction land, have become a serious phenomenon impeding agriculture intensification and scale economy [1, 2], thus impeding the modernization of agriculture. Many countries (see, e.g., for the USA [3, 4], Nepal [5]; Indonesia [6]; Pakistan [7]; and also multiple European countries [2]) have shed light on these issues and proposed various solutions (such as land policies and land management communities) to this problem. In China, the rural land circulation policy has been put forward as an effective solution to the fragmentation and outflow of cultivated land. Over the course of the introduction and refinement of this policy, the proportion of rural land circulation in China has risen from 2.6 percent in 1996 to 40 percent in 2018, and the total circulation scale has now reached 2.4 million hectares* [8].

However, the rural land circulation policy might induce farmers to change their original planting structure [9–11] and consequently impact national food security [11–14]. Recently, some scholars have raised concerns that rural land circulation might induce farmers to change their planting structure to avoid “grain orientation” [14–18], which would violate the fundamental original intention of the land circulation policy as strengthening food security. Thus, figuring out the effect of rural land circulation on agricultural planting adjustment is especially critical for countries like China, which accounts for a population of around 1.35 billion (almost one-fifth of the world’s population), with the burden of food demand relying on 230 million of the 260 million agricultural laborers.

Ahead of the unsolved dispute on the effect of rural land circulation on agricultural planting structure [14–21], some studies propose that the effect might vary according to different factors such as the scale of the circulated land [22, 23], the subsidies that governments use to stimulate farmers’ preferences for growing food crops [24] or the mobility of the agricultural labor force [25, 26]. However, previous studies have been unable to cover all of the mentioned factors within one model, partly due to the problems of self-selection and endogeneity when including those variables in the model). Furthermore, land conditions, such as geographical location, soil condition, and water condition, which may have a strong influence on the input and output mode of planting [27], have not been investigated to date. Thus, paddy land circulation and dry land circulation could possibly have different impacts on planting structure; however, these effects have not been explored in detail.

Based on the above considerations, this study aims to form a synthesis and detailed view of the impact of land circulation on agricultural planting structure adjustment. PSM (Propensity Score Matching) and GPSM (General Propensity Score Matching) methods are employed to partly solve the self-selection and endogeneity problems. It is believed that the investigation of this issue is of great significance for formulating a more reasonable land policy and implementing the national food security strategy.

The remainder of this paper is organized as follows. The second section offers a brief introduction to China’s rural land circulation policy and its relationship with crop planting structure adjustment. The third section introduces the methodology of combining PSM and GPSM. Results of the analysis are provided in the fourth section, and a brief conclusion and discussion are provided in the fifth section.

## Research background

### Rural land circulation in China

Cultivated land is the most irreplaceable productive material in agricultural production [28]; it is also an essential source of economic revenue for farmers, and has a pivotal influence on the country’s economic growth and social development [27, 29, 30]. However, urbanization and industrialization have tremendously changed the structures of land use, e.g., through the phenomena of cultivated land outflow and cultivated land fragmentation, which have been significant concerns attracting much academic concern in various countries [1, 2].

Cultivated land loss has been an intense problem in China after 1978 as a result of the rapid economic development and the consequent impact on the country’s land use [31–33]. Cultivated land has been lost due to several reasons: 1) Urban sprawl, i.e., the continued migration of homeowners out of cites to relatively inexpensive land and housing in the urban fringe [3, 34]; urban-rural land conversion, i.e., the increasing conversion of agricultural land to non-agricultural land (e.g., industrial construction and mining land) [3, 33, 35]; rural land fragmentation, caused by the continual expansion of rural construction land such as for building residences, and the inefficient planning of cultivated land which may develop into isolated cultivated plots, and cultivated land abandonment [3, 36–39]. The loss of cultivated land can lead to multiple problems that may affect farmers’ income and revenues [27, 35, 38, 40]. Furthermore, because a considerable proportion of lost cultivated land was used to grow food crops, this has resulted in a dramatic decrease in the production of grain, thus severely challenging national food security [27, 41, 42].

To resolve the challenging issue of cultivated land loss, China has continually improved its land policies. Since 2002, the land consolidation policy has aimed at restructuring agricultural resources in rural China through two kinds of measures. 1) measures controlling the urban-rural land conversion: this policy aims to maintain an “increasing vs. decreasing balance” of construction and cultivated land between urban and rural areas under the following rules: new construction areas must be smaller than old construction sites, and the area and quality of farmland reclaimed by old construction should be larger than farmland new constructions; 2) measures promoting the intensification and scale-operation of modern agriculture: community-based agricultural land consolidation projects (these communities are called “agricultural land share cooperative”) were developed to pool fragmented land and lease it as consolidated plots or employ laborers to farm the land [35, 36]. To date, the policy has been successful. The rapid expansion of rural land circulation has led to the enhancement of the agricultural socialization service level: there are more than 1.15 million service organizations at present, covering services for all aspects of agricultural production. Besides, multiple agricultural service innovations such as whole hosting, substitute cultivation (the outsourcing of agricultural work), and combined farming (multiple households form an agricultural community for co-production of crops), have emerged, which not only greatly improve the agricultural labor productivity, land productivity, and resource utilization, but also decrease the cost of production of agriculture [29, 41, 42].

### Relationship between rural land circulation and planting structure

China’s agricultural planting structure has undergone significant changes from the end of the 20th century till now. After China’s reform and opening-up policy, its multi-structure of agricultural planting gradually replacing the uni-structure of planting dominantly grain crops: Data of the grain sown area and its percentage of the total sown area showed a continuous fluctuation yet a general downward trend from 1978 to 2002 [43–45]. However, since 2003, both the grain sown area and its percentage of the total sown area began to rise due to the abolition of the agricultural tax policy, letting off the restrain of the grain planting capacity [44, 45]. In general, the year 2003 is a threshold that before it the agricultural planting structure in China oriented towards “non-grain” while after that year, the “grain-orientation” in agricultural planting structure emerged and has become an explicit trend [32].

Rural land circulation, ever since its operation, has become a potentially essential perturbation of agricultural planting structure and food security as well. Since cultivated land comprises food-crop land (grain land) and land on which cash crops are grown (non-grain land), thus food security depends mainly on the food-crop land (used for growing grain, hereafter known as “grain land”). However, rural land circulation, on the one hand, enlarges the cultivated land area (which might increase both the grain land and the non-grain land); on the other hand, it might induce farmers to change their planting structure, i.e., the ratio of growing food crops versus growing cash crops.

Farmers make their planting arrangements and choose whether to grow food crops or cash crops mainly according to the comparative cost and income from growing the different crops [9–11]. The land circulation will generate scale economy; however, the effect size of growing food crops and cash crop might differ [11]. Based on these considerations, several scholars have expressed concerns that the land circulation policy might be diverted away from its original purpose of improving grain production and facilitating food security, to favoring growing cash crops over food crops [14, 15]. They point out that the underlying reasons include a low efficiency of grain cultivation [16], leading to a negative difference in income between growing food crops and cash crops [18]; the inadequacy of grain subsidies that local governments offer to farmers [15]; and the relatively high cost generated by land circulation contracts [35, 46].

Despite these scholars’ concerns, other studies offer the viewpoint that land circulation does not necessarily lead to a shift away from the “grain orientation” of planting structures [19, 20, 21]. These studies have mostly found land circulation maintains the “grain orientation” by changing the rural labor orientation, which then fertilizes flexible solutions for growing food crops while in the meantime improving the household income. For instance, land circulation promotes the development of agricultural outsourcing services [21], so that few laborers are needed for the food crops planting, which consequently releases the agricultural labor into the non-agricultural job positions [19]. A new trend of “crop planting + non-agricultural work” mode is forming among the rural residents: A property of rural farmers now are transferred into part-time farmers/workers, and benefit from this new arrange of labor resources with a rise in the household income [19, 20], which maintains their decision of food crop planting instead of investing more efforts into planting cash crops. A substantial study was made to find that the higher developing level of agricultural outsourcing services, the higher level of the household labor income, together with the longer spatial distance for rural residents to get from their farms to their non-agricultural workplace (which could crowd out their labor investment into the crop planting work), the stronger intention that they will remain planting food crop [21].

The third school of thought holds the opinion that the relationship between land circulation area and planting structure adjustment is not completely linear, but “U-shaped”, given that many factors potentially mediate this relationship [22]. The most discussed factor is the scale of the land. For example, [22] found that although small-scale farmers could possibly switch from growing food crops to cash crops, the grain planting area and production of large-scale farmers remained stable; [23] similarly found that small-scale farmers will turn to cash crops (which is also consistent with [24]), whereas large-scale farmers will increase their planting of food crops. Correlated with the rural labor force transfer, [23] found that the effect of agricultural labor force transferring into non-agricultural duties on the planting structure adjustment relied on planting scales: local non-agricultural employment would reduce the grain planting proportion of small-scale farmers, while migrant workers would increase the proportion of their grain-planting activity; local non-agricultural employment will increase the proportion of the unit of wheat and corn planting and grain output for large-scale farmers, while migrant work will reduce the proportion of grain planted. Moreover, considering there are different kinds of food crops, some of which are perceived more beneficial when planting by farmers than the others [41], thus quite a few studies claimed that land circulation promoted an adjustment of planting structure by causing farmers to plant a different food crop aside from their original choice (an adjustment within the scope of food crops) [20, 46], rather than to plant cash crops (which could be an adjustment between the food crops and the cash crops). Consequently, the total orientation of food crops could stay untouched. Substantial studies showed that the land circulation would increase the proportion of grain planting only by increasing the proportion of rice planting, while the proportion of wheat and maize remain unchanged [20]. Besides, some geographical factors were also indicated by several studies to be included into consideration, e.g., Zhang and Jiang [47] pointed out that rural land circulation had an inconsistent effect on the “grain-orientation” of farmers in different Chinese regions because of differences in land conditions.

It could be implied that the effect of rural land circulation on planting structure requires more in-depth and synthetic investigations to reach a consensus. To date, models that include all the factors mentioned above are lacking. This limitation may result from the concerns of generating endogeneity and difficulties in identifying suitable instrument variables. Furthermore, we argue that such a model could easily generate a self-selection problem that might reduce the accuracy of the analyzed results. Moreover, extant research does not distinguish the different effects of different types of land circulation on planting structure adjustment, which, in our view, would provide a comprehensive understanding of this issue, together with more practical insights for the improvement of agricultural policies and modern agriculture development.

## Data and methods

### Data sources

The data used in this study were collected through a field survey of farmers in China. This study was approved by the ethics committee of the Jiangxi University of Finance and Economics, and conformed to the ethical standards for conducting research established by the National Advisory Board on Research Ethics of China. This survey was conducted in Jianli County and Qichun County in Hubei Province in the year 2018. More speculate, Jianli County belongs to the city of Huanggang, and is located in the south of Hubei Province, with a population of 1 571 400 and an elevation ranging from 23.5 to 30.5 feet; its total area is 320 100 hectares, of which is the 1 762 300 hectares of farmland [48]. Qichun County, belonging to the city of Jingzhou, is located in the southeast of Hubei Province with a population of 1 011 100 and an elevation ranging from 39.4 to 4 080.6 feet; its total area is 239 836 hectares, including 67 164 hectares of farmland [49].

The survey covered 44 villages in 11 towns; 26 rural households were selected randomly from each village. Thus, a total of 1 144 rural households were surveyed. Researchers achieved the verbal consent (which was recorded by a voice recorder) of each respondent before the survey was conducted. The survey is anonymous. The questionnaire (refer to S1 File) included series of questions to obtain basic information on the farmers’ family size, production resource endowment, material assets, production arrangements, and their perception and behaviors in relation to land circulation. A total of 1 120 valid samples (data is provided in S2 File) were obtained after eliminating 24 invalid responses.

#### Description of variables

*Agricultural planting structure*. Agricultural planting structure can be measured as “grain orientation,” which is the ratio of sown area of grain crops to the total sown area [15, 26], or “non-grain orientation,” the ratio of sown area of cash crops to the total sown area [33, 47]. Considering that the former is used more often in land cultivation research, this study relied on the former measurement to represent the structure of agricultural planting.

*Land circulation*. This research mainly analyzes the impact of land circulation on agricultural planting structure based on two aspects: whether or not land circulation is practiced (under this circumstance, dummy variables were created) and the area of the land being circulated. Further, this study distinguishes the circulated land as paddy land (a flooded parcel of arable land used for growing rice and other semiaquatic crops [50]) or dry land (zones where precipitation) is balanced and the land can be used for growing wheat and other crops such as drought-tolerant crops [25]). Therefore, three dummy variables representing whether or not to circulate the land (total land, paddy land, or dry land) and three continuous variables representing the area of the circulated land (total land, paddy land, or dry land) are generated.

*The education level of the householder (abbreviated to “education”)*. The head of a household (the householder) is one of the important decision-makers of the whole family’s plans, especially in rural districts, when coming to the farming plans [43, 51], and the education level of the householder will directly affect the family’s decision-making in this context.

*The size of the household (abbreviated to “household size”)*. Labor force is a basic element of agricultural production arrangements: the availability of an abundant labor force can induce farmers to choose labor-intensive crop varieties for planting [52]. However, household size will also influence the rural-to-urban labor migration, which will impact the agricultural labor force [54]. Moreover, the food consumption is dependent on the size of the family [53], which will be taken into consideration when deciding on planting structures.

*The proportion of migrant workers (abbreviated to “migrant”)*. Migration of workers away from the farmer’s labor force will reduce the agricultural labor force of the family; thus, farmers may choose to use machinery to replace the lost labor force and consequently consider planting technology-intensive crops [54].

*The attitude towards agricultural subsidies (abbreviated to “subsidy”)*. Although Direct Grain Subsidy (China’s nation-wide yearly subsidies which have been paid by the local government since 2004 to farmers before the grain is sown depending on the area they plan to grow grain; for more details, see Gale [55]; Zhang and Chen [56]) and agricultural subsidies will induce farmers to plant more food crops, this effect is also dependent on farmers’ satisfaction with these subsidies [26, 29].

*The cost of food crop planting (abbreviated to “cost of food crop”)*. The cost of producing grains consisted of the cost of seeds, fertilizer, farm implements will influence the net benefit of planting food crops; thus reduction in such a cost could encourage farmers to grow food crops through an expectation of a higher planting income [11].

*The cost of cash crop planting (abbreviated to “cost of cash crop”)*. Similar to the effect of a reduction in food crop planting cost, reducing the cost of planting cash crops will increase the comparative benefits of cash crops, thus encouraging farmers to plant more of such crops [11].

*The level of agricultural mechanization (abbreviated to “mechanization”)*. Extant research has pointed out that the level of agricultural mechanization can effectively reduce agricultural production costs, save agricultural labor time, and even increase agricultural production capacity [36, 57, 58]. Compared with cash crops, the production of food crops requires more agricultural machinery [11]. Therefore, agricultural mechanization will presumably promote “food-crop orientation” of cultivated land to a certain extent.

*The proportion of farmland irrigation (abbreviated to “irrigation”)*. Water resources are an important element of agricultural production; however, the requirements of water resources by different types of crops are different [59, 60]. Thus, farmland irrigation will impact farmers’ planting decisions.

*The type of the terrain (abbreviated to “terrain type”)*. Terrain type is among the essential components of the production environment of agriculture [27, 61, 62]: inputs and outputs of crop planting can be quite different between plain land and mountain land (or high land) [2, 5]. Therefore, this study included the type of the land as a factor affecting farmers’ planting decisions.

All the variables mentioned above are summarized in Table 1.

**Table 1:** Definitions of the variables and the statistics.

Variables	Definition and Coding	Mean	Standard Deviation	Min	Max
Structure	The structure of agricultural planting (area of food-crop planting divided by the total crop-planting area)	0.7254	0.3780	0	1
Total land circulation (dummy)	Whether the land is circulated (1 = Yes; 0 = No)	0.1473	0.3546	0	1
Paddy land circulation (dummy)	Whether the paddy land is circulated (1 = Yes; 0 = No)	0.1036	0.3049	0	1
Dry land circulation	Whether the dry land is circulated (1 = Yes; 0 = No)	0.0725	0.2595	0	1
Total Land circulation area	The total area of circulated land (the actual area)	1.5119	4.8507	0	25
Paddy land circulation area	The area of the circulated paddy land (the actual area)	1.0433	4.0491	0	25
Dry land circulation area	The area of the circulated dry land (the actual area)	0.4807	2.5793	0	25
Education	The education level of the householder (the actual value)	6.8891	3.4650	0	15
Household size	The size of the household (the actual value)	4.1415	1.7805	1	10
Migrant	The proportion of migrant workers (the number of migrant workers divided by the number of households)	0.3829	0.3485	0	1.5
Subsidy	The attitude towards agricultural subsidies (1 = Satisfied; 0 = Not Satisfied)	0.7837	0.4120	0	1
Cost of food-crop	The cost of food-crop planting (average food-crop planting cost per hectare)	2.0522	2.8798	0	6.80
Cost of economic-crop	The cost of cash crop planting (average economic crop planting cost per hectare)	5.0635	1.7399	0	6.11
Mechanization	The level of agricultural mechanization (the sum of machine farming rate multiplied by 0.4 and machine seeding rate multiplied by 0.3)^a^	0.7180	0.1329	0.50	1
Irrigation	The proportion of farmland irrigation (the effective irrigation area divided by the cultivated land area)	0.5430	0.2220	0.05	1
Terrain type	The type of land (1 = Plain; 0 = Non-plain)	0.3326	0.4714	0	1

^a^ The level of agricultural mechanization is calculated according to the guidelines provided by the Ministry of Agriculture and Rural Affairs of China.

#### Methodology

Using land circulation variables to regress the agricultural planting structure directly may lead to the loss of efficiency of model estimation because of problems of sample self-selection or endogeneity. Theoretically, there may be a causal relationship between land circulation and agricultural planting structure: Land circulation will produce a scale operation effect due to the expansion of the area, thus will farmers’ decisions on planting structure. In addition, the initial conditions under which farmers participate in land circulation are not exactly the same as those under which farmers do not participate in land circulation, which may also lead to the problem of sample self-selection. The PSM method can effectively resolve the bias and endogeneity generated by sample self-selection [63]. This method employs a “counterfactual framework” [64], assuming that farmers participating in land circulation are the treatment group and farmers that do not participate in land circulation are the controlled group, and then matches the treatment group and the control group according to the propensity score value.

The operation of the PSM method is as follows: first, the conditional probability of land circulation for each sample under the condition of a given influencing factor eigenvector is estimated by a logit or probit model, which is the propensity score (PS). It is specifically expressed as follows:

$${\text{PS}}_{\text{i}}=\text{Pr}[{\text{D}}_{\text{i}}=1|{\text{x}}_{\text{i}}]=\text{E}[{\text{D}}_{\text{i}}=0|{\text{x}}_{\text{i}}\text{]}$$ (1)

In the above equation, D_i_ = 1 means that farmers participate in land circulation, D_i_ = 0 means that farmers do not participate in land circulation, and x_i_ refers to the observable characteristics of farmers. Then, different matching methods are used to match the cases in the control group and those in the treatment group: the matching methods used in this research include radius caliper matching, kernel density matching, local linear matching, and Markov matching [65, 66]. By using the control group to simulate the counterfactual state of the treatment group, the differences in the agricultural planting structure of the farmers under the two mutually exclusive conditions of land circulation and uncirculated land are compared. The difference is the net treatment effect. The average treatment effect (ATT) of the agricultural planting structure can be expressed as follows:

$$ATT=E[y_{1i}-y_{0i}|D_{i}=1]=\frac{1}{N_{t}}\sum_{i\in t}y_{t}^{i}-\frac{1}{N_{t}}\sum_{j\in t}\lambda (p_{i},p_{j})y_{c}^{j}$$ (2)

In formula (2), *N*_*t*_ refers to farmers practicing land circulation, t is the experimental group after matching and the controlled group before matching, $y_{c}^{i}$ is the observation value of the i-th land circulation farmer in the experimental group, $y_{c}^{j}$ is the observation value of the j-th farmer who has not circulated land in the controlled group, *p*_*i*_ is the prediction probability value of the experimental group farmer i, *p*_*j*_ is the prediction probability value of the controlled group farmer j, *λ*(*p*_*i*_,*p*_*j*_) is the weight function and different matching methods corresponding to different weight functions.

The PSM method finds the difference in the agricultural planting structure between land circulation households and land non-circulation households rather than conduct an accurate evaluation based on continuous variables. In order to capture the causal relationship between the land circulation area and the agricultural planting structure, and the logical relationship between the land scale management area and the agricultural planting structure, this study employs a generalized propensity score matching method (GPSM) [64]. This model resolves the sample self-selection bias and endogeneity problems effectively, and at the same time, can estimate the impact of independent variables on dependent variables at any processing level [67]. The operational concept of GPSM is as follows:

First, *G*(*T*_*i*_), the conditional probability distribution of continuous processing variable T, can be estimated by the maximum-likelihood method when the covariate X is given:

$$G(T_{i})|X_{i}∼N(y(\lambda X_{i}),\sigma^{2})$$ (3)

In formula (3), y(λX_i_) is the linear function of covariate X; λ and σ^2^ are the parameters to be estimated. The generalized propensity score was estimated according to the covariate X,

$${\hat{P}}_{i}=\frac{1}{\sqrt{2\pi {\hat{\sigma }}^{2}}}exp\left\{-\frac{1}{2{\hat{\sigma }}^{2}}[G(T_{i})-y(\lambda X_{i})]\right\}$$ (4)

Second, the processing variable T and the generalized propensity score ${\hat{P}}_{i}$ are estimated from formula (4) are used to construct the model; then, the conditional expectation (i.e., agricultural production cost) of the result variable *F*_*i*_ is obtained:

$$E({\hat{P}}_{i}|T_{i},{\hat{P}}_{i})=\gamma_{0}+\gamma_{1}T_{i}+\gamma_{2}T_{i}^{2}+\gamma_{3}{\hat{P}}_{i}+\gamma_{4}{\hat{P}}_{i}^{2}+\gamma_{5}T_{i}{\hat{P}}_{i}$$ (5)

In formula (5), the role of $\hat{P}$, ${\hat{P}}_{i}^{2}$, and $T_{i}{\hat{P}}_{i}$ is to eliminate the problems of endogeneity and sample selection bias in the model.

Finally, the regression result of formula (4) is substituted into formula (5); then, the expected value of the result variable *F*_*i*_ when the processing variable is t can be obtained:

$$\hat{E}[F(t)]=\frac{1}{N}\sum_{i=1}^{N}\left[{\hat{\gamma }}_{0}+{\hat{\gamma }}_{1}t+{\hat{\gamma }}_{2}t_{i}^{2}+{\hat{\gamma }}_{3}\hat{p}(t,X_{i})+{\hat{\gamma }}_{4}\hat{p}{\left(t,X_{i}\right)}^{2}+{\hat{\gamma }}_{5}t\hat{p}(t,X_{i})\right]$$ 6)

In formula (6), N is the sample observation value and $\hat{\text{p}}{\text{(t,X}}_{\text{i}}\text{)}$ is the conditional probability density prediction value of the processing variable. The value range of the processing variable $\bar{T}=[t_{0},t_{1}]$ is divided into n subintervals $\bar{T}=(n=1,2,\ldots ,n)$, in which the causal effect of land circulation area on agricultural planting structure can be estimated. By connecting the causal effects in each value range, we obtain a diagram of the functional relationship between the size of the causal effect and the land circulation area in the interval $\bar{T}=[t_{0},t_{1}]$.

## Results

### Test of sample matching effect

**Fig 1:**
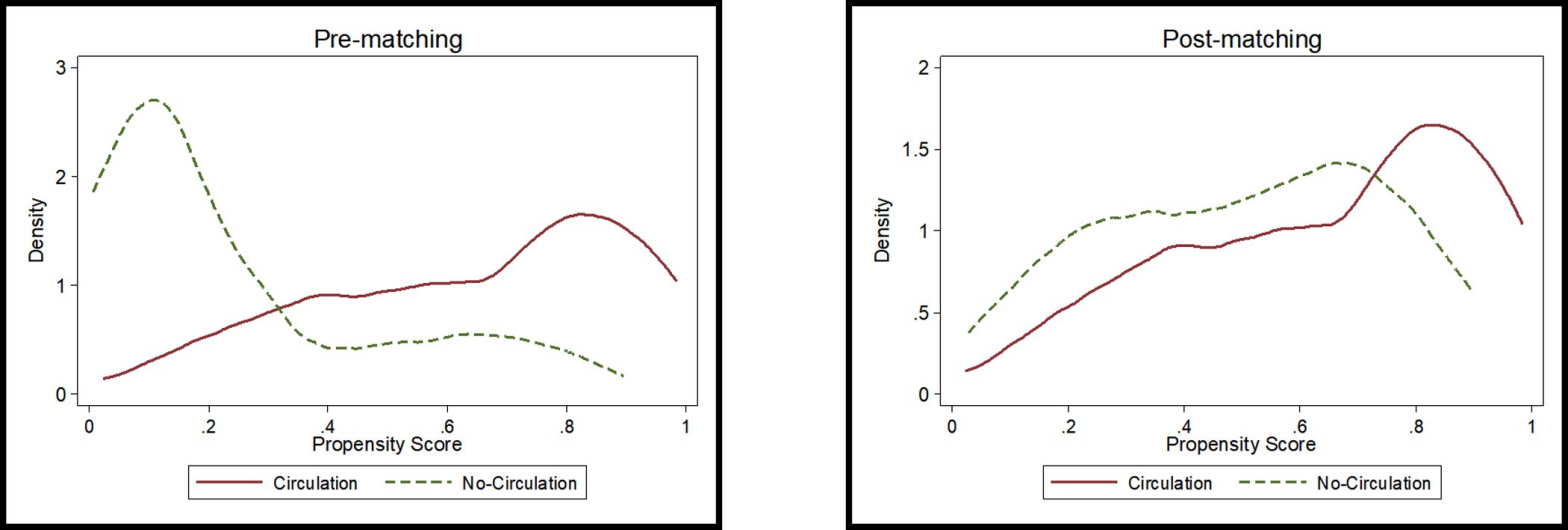
Probability distribution of propensity score nucleus before and after matching. (a) Probability distribution of propensity score nucleus before matching. (b) Probability distribution of propensity score nucleus after matching.

Before using PSM estimation, it is necessary to check the matching effect, or the balance of matching variables between the treatment group and the control group [68] by comparing the distribution of propensity scores before and after matching [69]. Fig 1 shows the PS values and kernel densities before and after the match for the total land circulation (treatment) group and the land is not circulated (control) group. It can be concluded that, as for the propensity scores, the treatment group and control group do not agree completely before matching; however, they tend to distribute similarly after matching. Thus the matching effect for the total land circulation is achieved. This paper also checked the matching effects for paddy land circulation and dry land circulation, respectively. Results show that both those matching effects are significant.

### PSM estimation of planting structure by land circulation

In order to compare the stability of the estimated results, different matching methods (as mentioned in the methodology section) were used to estimate the average treatment effect of land circulation, paddy land circulation, and dry land circulation on the agricultural planting structure, separately (as shown in Table 2).

**Table 2:** PSM estimation results of agricultural planting structure by land circulation (dummy).

Category	Matching method	Treatment group	Control group	ATT value^b^	SE^c^	T value
Whether the land is circulated	Not matched	0.8231	0.1985	0.6246***	0.0325	19.22
	Match 1	0.8146	0.4523	0.3623***	0.0564	6.42
	Match 2	0.7920	0.4367	0.3553***	0.0483	7.36
	Match 3	0.7920	0.4325	0.3595***	0.0576	6.24
	Match 4	0.7731	0.4163	0.3568***	0.0284	12.6
Whether the paddy land is circulated	Not matched	0.8583	0.2913	0.5671***	0.0417	13.58
	Match 1^a^	0.8583	0.4744	0.3839***	0.0540	7.11
	Match 2	0.8552	0.4701	0.3851***	0.0536	7.18
	Match 3	0.8552	0.4728	0.3824***	0.0672	5.69
	Match 4	0.8583	0.4644	0.3939***	0.0306	12.87
Whether the dry land is circulated	Not matched	0.7430	0.3781	0.3649***	0.0560	6.51
	Match 1	0.7402	0.6123	0.1278**	0.0633	2.02
	Match 2	0.7373	0.6277	0.1095*	0.0662	1.66
	Match 3	0.7373	0.6147	0.1225*	0.0668	1.83
	Match 4	0.7430	0.6025	0.1405***	0.0421	3.34

^a^ Matching 1–4 respectively indicate the radius caliper matching method, the kernel density matching method, the local linear matching method, and the Markov matching method;

^c^ ATT value = average treatment effects value;

^b^ SE = Standard error;

*, **, and *** represent the significance level of 10%, 5%, and 1% respectively.

It can be seen from Table 2 that the estimated results all pass the significance test. The average treatment effect of land circulation on agricultural planting structure indicates that land circulation significantly improves the agricultural planting structure by 0.62 units (reserving two decimals, and similarly hereinafter). The average treatment effects values estimated by the four matching methods are approximately 0.36 units, which means, in general, after eliminating the difference in samples by the matching method, the average coefficient of the net effect of total land circulation was 0.36. This result was significantly lower than the estimated coefficient before matching, indicating that sample estimation bias and endogeneity would indeed result in overestimation of the effect of land circulation. It can also be concluded that, compared with the circumstance without land circulation, the circumstance of land circulation can promote the agricultural planting structure towards a “grain orientation;” in other words, land circulation encourages farmers to grow more food crops. A possible reason for this effect is agricultural mechanization: the dependence on agricultural mechanization for growing food crops is stronger, which is more conducive to the operation of agricultural machinery operation during land circulation; further, the use of agricultural machinery to replace labor can reduce agricultural production costs. Moreover, this effect can be investigated from the perspective of multiple cropping index, which refers to the average number of crops planted on the cultivated land area in one year. An increase in the multiple cropping index would positively increase the unit production of the land, thus potentially improve farmers planting income. In the past, the improvement of the multiple cropping index was mainly by farmers planting cash crops aside from food crops on the land (their planting of food crops stayed constant, which would not contribute to the multiple cropping index); however, with the agricultural mechanization stimulated by land circulation, farmers now are prompt to plant addition rounds of food crops (e.g., districts inclining to plant one round of rice in the past change to plant two rounds of rice or to plant one round of rice added by one round of wheat) to improve their planting income. In addition, the offering of direct food subsidies for farmers who cultivate food crops will directly promote the grain-orientation of arable land.

Similarly to the circumstance of total land circulation, the average treatment effect values for paddy land circulation and dry land circulation before matching are 0.57 and 0.36, which, compared to the values of 0.39 and 0.13 after matching, declaring an overestimation of the effects. However, the average treatment effect values after matching show that both the paddy land circulation, and the dry land circulation, separately, can promote the agricultural planting structure towards a “grain orientation.” It can also be concluded that the average treatment effect of paddy land circulation is three times that of dry land circulation, indicating that paddy land circulation can promote the “grain orientation” of farmers’ planting structure better than dry land circulation. This may be because paddy plots are inherently more suitable for planting food crops; further, paddy plots can increase the grain-sown area by increasing the multiple cropping index, thus increasing the proportion of the sowing area for cultivated grain crops.

### GPSM estimation of land circulation area to planting structure

Before using GPSM analysis, we test whether the processing variables meet the supposed conditions of normal distribution. By testing the skewness and kurtosis of the distribution of land circulation area, paddy land circulation area, and dry land circulation area, it is found that the three processing variables obey the original hypothesis of normal distribution. Then, the fractional logit model is used to estimate the generalized propensity score to test whether the covariates adjusted by the generalized propensity score pass the balance test. The samples need to be grouped according to the idea of the balance test of Hirano and Imbens [64]. The value of the processing variable (the land circulation area) after extremum is [1]; therefore, according to the principle of subdividing the treatment intensity between the smaller areas and roughly dividing the larger treatment intensity range, the selected critical points are 0.3 and 0.7 of the treatment intensity. In accordance with the critical value, this paper divides the three types of land circulation samples into three groups, and assesses the balance by comparing the difference between the average value of a certain covariate in any group and the average value of the covariate after the combination of the other two groups. Table 3 summarizes the estimated values of the covariates in each group of the three land circulation samples. The estimated results show that after the adjustment of the generalized propensity score, none of the covariates are significant, indicating that the matching effect is good and has passed the balance test.

**Table 3:** GPSM estimation and balance test results of agricultural planting structure by land circulation area.

Variable	Total Land circulation area			Paddy land circulation area			Dry land circulation area		
	[0,0.3]	(0.3,0.7]	(0.7,1]	[0,0.3]	(0.3,0.7]	(0.7,1]	[0,0.3]	(0.3,0.7]	(0.7,1]
Education	-0.4714	-0.0175	0.1060	-0.5591	0.3190	0.2365	-0.1340	0.4146	-1.1103
	(0.3433)	(0.4682)	(0.3831)	(0.3417)	(0.4906)	(0.4746)	(0.4985)	(0.5808)	(0.7526)
Household size	0.0079	-0.0019	0.0077	-0.0110	0.0055	0.0158	0.0455	-0.0289	0.0089
	(0.0249)	(0.0314)	(0.0267)	(0.0254)	(0.0352)	(0.0329)	(0.0328)	(0.0359)	(0.0480)
Migrant	0.1289	-0.1542	-0.1742	0.1401	-0.0897	-0.1351	0.4763	-0.5527	-0.7576
	(0.1581)	(0.2150)	(0.1825)	(0.1855)	(0.2520)	(0.2441)	(0.1922)	(0.2308)	(0.3014)
Subsidy	0.0577	-0.0017	0.0282	0.0209	-0.1238	0.0820	0.0045	-0.0227	0.0033
	(0.0766)	(0.1037)	(0.0834)	(0.0775)	(0.1101)	(0.0981)	(0.0969)	(0.1081)	(0.1473)
Cost of food-crop	0.0369	-0.0358	-0.0139	0.0434	-0.0262	-0.0178	0.0032	-0.0080	0.0811
	(0.0222)	(0.0284)	(0.0175)	(0.0240)	(0.0418)	(0.0242)	(0.0424)	(0.0489)	(0.0640)
Cost of economic-crop	-0.0378	0.0933	-0.0047	-0.0949	0.0902	0.0901	-0.0577	0.0449	-0.0770
	(0.1020)	(0.1545)	(0.0989)	(0.1429)	(0.1723)	(0.1742)	(0.0541)	(0.0696)	(0.1047)
Mechanization	0.0486	-0.1179	-0.0880	0.0838	-0.1547	-0.0270	0.0987	-0.0617	-0.3144
	(0.0561)	(0.0812)	(0.0617)	(0.0688)	(0.1000)	(0.0927)	(0.0849)	(0.0991)	(0.1332)
Irrigation	0.0005	0.0811	-0.1009	0.0405	-0.0069	-0.0659	-0.0034	0.0115	0.0125
	(0.0423)	(0.0592)	(0.0471)	(0.0493)	(0.0705)	(0.0623)	(0.0601)	(0.0720)	(0.0964)
Terrain type	-0.0357	0.0925	-0.0266	-0.0096	0.0621	-0.0318	0.0441	0.0329	-0.1212
	(0.0439)	(0.0615)	(0.0475)	(0.0398)	(0.0603)	(0.0489)	(0.0606)	(0.0671)	(0.0945)

Then, the conditional expectation of the resulting variable agricultural planting structure in the three types of land circulation samples is estimated, and the second-order approximation estimation method is used to better fit the agricultural planting structure. The estimated results are shown in Table 4. The results show that, as for the total land circulation, the circulation area and its square, propensity score variable and its square, and their interaction items all passed the significance test; also, as for the paddy land circulation, the circulation area and its square, tendency score variable, and their interaction item passed the significance test; as for the dry land circulation, the circulation area and its square, tendency score variable and its square, passed the significance test. Those results indicate endogeneity exists as for all three types of land circulation.

**Table 4:** Second-order approximation estimation results for agricultural planting structure.

Variable	Total land circulation area		Paddy land circulation area		Dry land circulation area	
	coefficient	SE^a^	coefficient	SE	coefficient	SE
*T*^b^	1.9708***	0.2015	2.0437***	0.2670	1.3384***	0.4110
*T*^2^	-1.3429***	0.2031	-1.4447***	0.2997	-1.2136***	0.4335
*P*^c^	2.0116***	0.4581	1.4925***	0.5270	5.0395***	1.0344
*P*^2^	-1.8605**	0.8044	-1.4678	1.2115	-16.1675***	4.8050
*T* × *P*	-0.8042**	0.3769	-1.0634*	0.5873	-0.7133	2.1840
cons	0.0534	0.0513	0.2689***	0.0399	0.3026***	0.0397

^a^ SE = Standard error;

^b^ T = land circulation area;

^c^ P = propensity score.

After excluding the insignificant variables, the significant variables were substituted into Eq (6) to estimate the expected value and marginal change of agricultural planting structure of different types of land circulation areas at different treatment levels. To ensure the validity of the estimation results, we estimate the processing effect function of each GPSM by bootstrapping 500 times. According to the results shown in Table 5, the treatment effects of land circulation area, paddy land circulation area, and dry land circulation area are always positive; the relationship between land circulation area and agricultural planting structure is not linear; the treatment effects of the three types of land circulation increase with the increase in the circulation area, but the marginal effects of different types of land circulation are different at different treatment levels.

**Table 5:** GPSM treatment effect estimation results of agricultural planting structure by land circulation area.

T value	Total land circulation area		Paddy land circulation area		Dry land circulation area	
	Coefficient	SE^a^	Coefficient	SE	Coefficient	SE
0.1	0.3751***	0.0339	0.4363***	0.0228	0.0862***	0.0312
0.2	0.4128***	0.0473	0.4831***	0.0334	0.2344***	0.0404
0.3	0.6161***	0.0485	0.7045***	0.0375	0.571***	0.0434
0.4	1.096***	0.0494	1.1004***	0.0369	0.9851***	0.0515
0.5	1.6709***	0.0342	1.8094***	0.0662	1.5197***	0.0658
0.6	2.416***	0.0314	2.7112***	0.0938	2.22***	0.0847
0.7	3.3357***	0.0297	3.8013***	0.1303	3.086***	0.0971
0.8	4.4299***	0.0301	5.0799***	0.1766	4.1176***	0.0940
0.9	5.6987***	0.0371	6.5468***	0.2365	5.3149***	0.0713
1	7.1421***	0.0577	8.2021***	0.3164	6.6779***	0.0573

^a^ SE = Standard error.

More specifically, the effects of the total land circulation on agricultural planting structure are significant yet relatively small at the treatment level of 0–0.3 (0.38 for 0.1 level; 0.41 for 0.2 level; 0.62 for 0.3 level); when exceeding the 0.3 treatment level, the effects accelerate in strength (e.g., 1.10 for 0.4 level; 1.67 for 0.5 level; 2.42 for 0.6 level). The effects of the paddy land and dry land circulation obey a similar trend. On the whole, there are increasing returns to scale effect of the total land, paddy land, and dry land circulation on agricultural planting structure; that is, the increasing speed for the food crop planting area is higher than that for the area of either land circulation type. The possible reason for this phenomenon is that land circulation has a certain threshold effect on farmers’ adjustment of agricultural planting structure. It is only when the land circulation area exceeds this threshold value that the effect of land scale operation on the improvement of agricultural planting structure becomes obvious, that is, in the case of small circulation scales, the change in different crop income ratio caused by agricultural scale operation is not sufficient to induce the readjustment of agricultural production structure by farmers’ households.

Additionally, at any treatment level, the effect of increasing the circulation area of paddy lands on the adjustment of agricultural planting structure is greater than that for dry land circulation, which indicates that paddy lands can promote farmers’ adjustment of agricultural planting structure to “grain orientation.” There are three possible reasons: first, land circulation benefits scale management and the grain direct subsidy policy has a greater effect on the grain productivity. Second, there is more space for the improvement of the multiple cropping index of paddy lands, such as the “single to double” mode of rice; third, land circulation reduces the cost of production of food crops in many ways. For example, land circulation can promote the mechanization of grain crops; labor costs can be reduced by using machinery instead of agricultural labor. However, the available types and rates of utilization of agricultural machinery for cash crops are not high, which makes it difficult for cash crops to adopt agricultural machinery on a large scale after land circulation, which is not conducive to the adjustment of planting structure towards growing cash crops. Moreover, after land circulation, farmers’ land preparation activities will greatly increase the protection of land and promote the generation of high-standard farmland, which will provide the possibility for agricultural production to reduce costs and increase production.

## Conclusions, implications, and future research

### Conclusions

With the continuous increase in the scale of rural land circulation, it has become crucial to address the adjustments, i.e., the planting structure decisions, that farmers make in the context of this land policy. Will rural land circulation sway planting decisions towards a “non-grain orientation,” or will rural land circulation instead facilitate a “grain orientation” of crop planting? Based on field survey data for farmers in Hubei province, China, in 2018, this study used PSM and GPSM methods to address the influence of circulation of different types of rural land on farmers’ planting structure decisions. The results support the latter inference by indicating that compared with the absence of land circulation, land circulation can significantly promote the adjustment of farmers’ agricultural planting structure to the direction of “grain orientation.” Land circulation supports its original purpose of improving food crop production and facilitating food security. Furthermore, this study confirms this finding by pointing out that circulation of paddy lands and dry land can both promote the “grain orientation” of planting structure; however, the effect of paddy land circulation is much stronger than that of dry land circulation. Additionally, adjustment of the “grain orientation” of the planting structure is obviously enhanced with an increase in the area of rural land under circulation, which means an increased return to scale. These findings contribute to the extant research on the practice of rural land circulation.

### Implications

These findings offer a more in-depth understanding of how rural land circulation influences farmers’ planting structure adjustment, which will address this disputed issue to some extent. Findings of this study divert from those of previous studies supporting a negative impact of rural land circulation on “grain orientation” [14–18] of planting, but are consistent with those such as Luo and Qiu [19], Qian [20], Sun and Su [21] in advocating that land policy could facilitate more “grain-orientated” crop planting in China. This study confirms that view via a more valid methodology involving combining PSM and GPSM methods to avoid problematic self-selection and endogeneity. Thus, this study could provide a synthesis model discussing multiple correlated factors relevant to the effect of rural land circulation on planting structure. Besides, by using both PSM and GPSM methods, the status of rural land circulation can be measured both as a dummy variable and numerical variables for different types of land. Therefore, this study offers a perspective of discussing details related to land types in rural land circulation.

In regard to practical implications, the findings confirm the validity of China’s rural land circulation policy, which we believe provides valuable direction for other countries to better address their land problems and develop modern agricultural methods. For instance, as for developing countries with land systems similar to China’s and intend to conduct similar land circulation activity, generating “non-grain orientation” of agricultural planting structure change and consequently impeding national food security seems not to become the main concern.

Furthermore, this study compares the effect of circulation of different types of land, and the finding implies that, at least in China (a country that grows vast amounts of food crops such as rice in paddy lands), circulation of paddy lands should be emphasized to better facilitate the “grain orientation” of planting structure and thus to facilitate food security. One possible explanation might be that paddy-field grain crops such as rice could sell for a better price per hectare than dry-land crops such as wheat; therefore, growing grain crops in paddy lands is more “economical” and thus could offer more powerful stimulation for planting than growing grain crops on dry land.

However, it is also necessary to facilitate congruency of these correlated factors to enhance crop production and increase food security. For example, food crop production costs could be reduced by the economy of scale effect of farming mechanization. To achieve farming mechanization, farming communities should be authorized and further developed and strengthened [29]. Operational experience and management architectures from non-agricultural rural communities such as the township village enterprises [70] could be employed to improve the operation and management of such agricultural rural communities. Besides, excessive land circulation cost will increase the total farming cost, and affect farmers’ enthusiasm for growing crops [26]. Thus, a more flexible market regulation mechanism is expected to promote the scale of rural land transfer and, furthermore, decrease the land circulation costs. Based on this consideration, the government should formulate guide prices for different types of land circulation, establish competitive farmland in contract with the appropriate trading market, and explore the construction of a low-cost farmland circulation mechanism.

More importantly, because the agricultural subsidy has been found to exert significant influence on farmers’ planting decisions [26, 55], this study implies that it is critical to continue to execute subsidies for large-scale grain cultivation. Moreover, subsidies should be more adaptive to the local agricultural conditions, and should be updated according to the nation’s agricultural strategies. Additionally, the findings also imply a great value for the governments of countries engaged in the development of modern agriculture to enhance food security via the following: improvement in the level of socialized agricultural services [71], popularization of high-yield and high-quality grain varieties [72, 73], and investment in agricultural scientific research to effectively reduce grain production costs.

### Limitations and future research directions

Although this study addresses the possible problem of measurement using an empirical model to explore the influence of the circulation of different land types, there remain limitations owing to constrained data sets: First, this paper did not discuss the influence of the circulation of non-cultivated agricultural land, e.g., woodland and grassland, on the agricultural planting structures, considering non-cultivated agricultural land is rarely owned and operated by normal farm households in the sampling districts. However, future research can engage in a larger scope consisting of multiple forms of circulated land. Second, this study mainly focuses on the effects of rural land circulation on the “grain versus non-grain orientation”, i.e., the proportion of grain crops and cash crops, and thus offers no further analysis from the perspective of crop variety. Based on the consideration that the scale economic benefit might be different among different crops, whether and how the land circulation will influence the variety of crops may be an issue worthy of discussion. Third, the data used in this study were cross-sectional field-survey data from farmers. Agricultural production is usually continuous, and the farmer’s planting decisions rely on the history of planting outcomes of previous years and also their outcome expectations in subsequent years [74, 75]. Under this circumstance, continuous panel data could facilitate a more accurate analysis of the impact of rural land circulation on the planting structure. Fourth, agricultural production is cyclical, and the growth cycles of cash crops and food crops are significantly different [76]. The duration of the land transfer contract would likely affect farmers’ planting decisions; thus, further consideration of the duration of land transfer contracts will enhance the reliability of the research results.

## Supporting information

S1 File Survey questionnaire. (DOC)

S2 File Sample data. (XLSX)
