# Supplementary material for: Will land circulation sway “grain orientation”? The impact of rural land circulation on farmers’ agricultural planting structures
Source: PLoS One. 2021 Jun 24;16(6):e0253158. doi: 10.1371/journal.pone.0253158 (PMC8224926; doi:10.1371/journal.pone.0253158)
Supplement: S1 File — (DOC) [file pone.0253158.s001.doc]

**Survey questionnaire**

***original Chinese version***

农户编码

**中国农户经济行为调查问卷**

| 省 |  | | |
| --- | --- | --- | --- |
| 县 |  | | |
| 镇/乡 |  | | |
| 村 |  | | |
| 户主姓名 |  | 对应编码 |  |
| 被调查人 |  | 对应编码 |  |
| 调查人姓名 |  | | |
| 调查日期 |  | | |

**A 家庭人口信息**

|  | **个人编码** | **01户主** | **02** | **03** | **04** | **05** | **06** | **07** | **08** | **09** | **10** |
| --- | --- | --- | --- | --- | --- | --- | --- | --- | --- | --- | --- |
| 01 | 与户主关系 （1户主；2配偶；3 儿子；4儿媳；5女儿；6 女婿； 7孙子/女 ；8父/母 ；9祖父/祖母；10＝其它） |  |  |  |  |  |  |  |  |  |  |
| 02 | 性别 ( 1男；2女) |  |  |  |  |  |  |  |  |  |  |
| 03 | 年龄 （岁） |  |  |  |  |  |  |  |  |  |  |
| 04 | 民族 （1 汉族 2其它） |  |  |  |  |  |  |  |  |  |  |
| 05 | 婚姻状况 （ 1未婚 2初婚 3 再婚 4离婚 5丧偶） |  |  |  |  |  |  |  |  |  |  |
| 06 | 受教育水平（1 未入学 2小学未毕业 3小学 4 初中 5 高中或中专5 大专及以上；填写毕业水平） |  |  |  |  |  |  |  |  |  |  |
| 07 | 主要职业（（16 岁以上）1 农业经营；2 非农经营；3 专业技术人员；4 企事业单位管理者；5企事业单位一般工作人员；6 工人；7 服务行业人员；8 学生；9 其它） |  |  |  |  |  |  |  |  |  |  |

**B家庭耕地资产**

| 土地资源 | 面积（公顷） |
| --- | --- |
| 承包耕地 |  |
| 其中：水田 |  |
| 旱地 |  |
| 转入耕地 |  |
| 其中：水田 |  |
| 旱地 |  |
| 转出耕地 |  |
| 其中：水田 |  |
| 旱地 |  |
| 机耕面积 |  |
| 机播面积 |  |
| 机收面积 |  |
| 灌溉面积 |  |

**C 家庭农业经营行为**

种植业

| 品种 | 面积  （公顷） | 总产量  （斤） | 出售量（斤） | 价格  （元/斤） | 总产值  （元） | 投入（元） | | | | | |
| --- | --- | --- | --- | --- | --- | --- | --- | --- | --- | --- | --- |
|  |  |  |  |  |  | 种子 | 农药  化肥  农膜 | 机械  燃料  电力 | 雇工 | 其他 | 合计 |
|  |  |  |  |  |  |  |  |  |  |  |  |
|  |  |  |  |  |  |  |  |  |  |  |  |

**D 农业支持政策**

| 01 | 您对农业补贴的态度？ 1.满意 2.不满意 |  |
| --- | --- | --- |

***Translated English version***

Farmer ID

**Questionnaire on economic behavior of peasant households in China**

| Province |  | | |
| --- | --- | --- | --- |
| County |  | | |
| Town/township |  | | |
| Village |  | | |
| Name of the household head |  | Relative ID |  |
| Respondent |  | Relative ID |  |
| Name of the respondent |  | | |
| Date of survey |  | | |

**A Information of the household population**

|  | **Personal ID** | **01** head of household | **02** | **03** | **04** | **05** | **06** | **07** | **08** | **09** | **10** |
| --- | --- | --- | --- | --- | --- | --- | --- | --- | --- | --- | --- |
| 01 | Relationship with head of household (1 head of household; 2 a spouse; 3 son; 4 daughter-in-law; 5 daughters; 6 son-in-law; 7 grandchildren/children; 8 Father/mother; 9 Grandfather/grandmother; 10 Others) |  |  |  |  |  |  |  |  |  |  |
| 02 | Gender (1 male; 2 female) |  |  |  |  |  |  |  |  |  |  |
| 03 | Age (years) |  |  |  |  |  |  |  |  |  |  |
| 04 | Nationality (1 Han; 2 Others) |  |  |  |  |  |  |  |  |  |  |
| 05 | Marital status (1 unmarried; 2 first marriage; 3 remarriage; 4 divorced; 5 widowed) |  |  |  |  |  |  |  |  |  |  |
| 06 | Education level (1 not enrolled in school; 2 not graduated from primary school; 3 primary school; 4 junior high school; 5 senior high school or technical secondary school; 5 junior college or above; Fill in the graduation level) |  |  |  |  |  |  |  |  |  |  |
| 07 | Main occupation (for age 16 and above: 1 Agricultural operation; 2 Non-agricultural operation; 3 Professional and technical personnel; 4. Managers of enterprises and institutions; 5. General staff of enterprises and institutions; 6 Workers; 7. Personnel in the service industry; 8 students; 9 Other) |  |  |  |  |  |  |  |  |  |  |

**B Farmland assets of the household**

| Land resources | Area (Hectares) |
| --- | --- |
| Contracted farmland |  |
| *Among them:* Paddy land |  |
| Dry land |  |
| Circulated farmland: Roll in |  |
| *Among them:* Paddy land |  |
| Dry land |  |
| Circulated farmland: Roll out |  |
| *Among them:* Paddy land |  |
| Dry land |  |
| Mechanical-cultivated land |  |
| Mechanical-sown land |  |
| Mechanical-harvested land |  |
| Irrigated land |  |

**C Agricultural management behavior of the household**

Planting

| Variety | Total area (Hectares) | Total yield  (Jin) | Sales quantity (Jin) | Unit price  (RMB yuan/Jin) | Gross output value (RMB yuan) | Investment (RMB yuan) | | | | | |
| --- | --- | --- | --- | --- | --- | --- | --- | --- | --- | --- | --- |
|  |  |  |  |  |  | Seed | Pesticide, fertilizer, film | Machinery, fuel, electricity | Employment | Other | Total |
|  |  |  |  |  |  |  |  |  |  |  |  |
|  |  |  |  |  |  |  |  |  |  |  |  |

**D Agricultural support policy**

| 01 | What is your attitude towards agricultural subsidies? 1. Satisfied 2. Unsatisfied |  |
| --- | --- | --- |
